# Comparison of Clinical Study Results Reported in medRxiv Preprints vs Peer-reviewed Journal Articles

**DOI:** 10.1001/jamanetworkopen.2022.45847

**Published:** 2022-12-09

**Authors:** Guneet Janda, Vishal Khetpal, Xiaoting Shi, Joseph S. Ross, Joshua D. Wallach

**Affiliations:** 1 Yale School of Medicine, New Haven, Connecticut; 2 Department of Medicine, Warren Alpert Medical School of Brown University, Providence, Rhode Island; 3 Department of Environmental Health Sciences, Yale School of Public Health, New Haven, Connecticut; 4 Section of General Medicine and the National Clinician Scholars Program, Department of Internal Medicine, Yale School of Medicine, New Haven, Connecticut; 5 Center for Outcomes Research and Evaluation, Yale–New Haven Health System, New Haven, Connecticut; 6 Department of Health Policy and Management, Yale School of Public Health, New Haven, Connecticut; 7 Department of Epidemiology, Rollins School of Public Health, Emory University, Atlanta, Georgia

## Abstract

**Importance:**

Preprints have been widely adopted to enhance the timely dissemination of research across many scientific fields. Concerns remain that early, public access to preliminary medical research has the potential to propagate misleading or faulty research that has been conducted or interpreted in error.

**Objective:**

To evaluate the concordance among study characteristics, results, and interpretations described in preprints of clinical studies posted to medRxiv that are subsequently published in peer-reviewed journals (preprint-journal article pairs).

**Design, Setting, and Participants:**

This cross-sectional study assessed all preprints describing clinical studies that were initially posted to medRxiv in September 2020 and subsequently published in a peer-reviewed journal as of September 15, 2022.

**Main Outcomes and Measures:**

For preprint-journal article pairs describing clinical trials, observational studies, and meta-analyses that measured health-related outcomes, the sample size, primary end points, corresponding results, and overarching conclusions were abstracted and compared. Sample size and results from primary end points were considered concordant if they had exact numerical equivalence.

**Results:**

Among 1399 preprints first posted on medRxiv in September 2020, a total of 1077 (77.0%) had been published as of September 15, 2022, a median of 6 months (IQR, 3-8 months) after preprint posting. Of the 547 preprint-journal article pairs describing clinical trials, observational studies, or meta-analyses, 293 (53.6%) were related to COVID-19. Of the 535 pairs reporting sample sizes in both sources, 462 (86.4%) were concordant; 43 (58.9%) of the 73 pairs with discordant sample sizes had larger samples in the journal publication. There were 534 pairs (97.6%) with concordant and 13 pairs (2.4%) with discordant primary end points. Of the 535 pairs with numerical results for the primary end points, 434 (81.1%) had concordant primary end point results; 66 of the 101 discordant pairs (65.3%) had effect estimates that were in the same direction and were statistically consistent. Overall, 526 pairs (96.2%) had concordant study interpretations, including 82 of the 101 pairs (81.2%) with discordant primary end point results.

**Conclusions and Relevance:**

Most clinical studies posted as preprints on medRxiv and subsequently published in peer-reviewed journals had concordant study characteristics, results, and final interpretations. With more than three-fourths of preprints published in journals within 24 months, these results may suggest that many preprints report findings that are consistent with the final peer-reviewed publications.

## Key Points

**Question:**

What is the concordance among sample size, primary end points, results for primary end points, and interpretations described in preprints of clinical studies posted on medRxiv that are subsequently published in peer-reviewed journals (preprint-journal article pairs)?

**Findings:**

In this cross-sectional study of 547 clinical studies that were initially posted to medRxiv and later published in peer-reviewed journals, 86.4% of preprint-journal article pairs were concordant in terms of sample size, 97.6% in terms of primary end points, 81.1% in terms of results of primary end points, and 96.2% in terms of study interpretations.

**Meaning:**

This study suggests that most clinical studies posted as preprints on medRxiv and subsequently published in peer-reviewed journals had concordant study characteristics, results, and final interpretations.

## Teaser

This cross-sectional study of preprints compares the agreement in characteristics, results, and conclusions of clinical studies posted as preprints to medRxiv and subsequently published in any peer-reviewed journal.

## Introduction

Preprints, which are preliminary research reports that have not yet undergone peer review, have been widely adopted to enhance the timely dissemination of research across many scientific fields.^1,2^ The launch of the preprint server medRxiv has recently led to the increasing use of preprints in the clinical and health science research community.^3^ Although the COVID-19 pandemic has highlighted several benefits of preprints, including the rapid and open evaluation of research findings,^4,5,6^ concerns remain that early, public access to preliminary medical research has the potential to harm patients or public health practices by propagating misleading or faulty research that has been conducted or interpreted in error.^7^

Although not all preprints will be published in peer-reviewed journals, among those that are (preprint-journal article pairs), it is possible to examine the extent to which the studies’ design, results, and conclusions are changed as of publication. Although previous evaluations have provided some reassurance of the consistency between preprints and subsequent publications, these efforts have often focused on specific fields and journal types. For instance, evidence suggests that COVID-19–related preprint-journal article pairs are largely similar in terms of their abstracts, figures, and interpretations.^8,9^ Moreover, preprints of clinical studies posted on medRxiv and subsequently published in clinical journals with the highest impact factors (impact factor >10) were found to have high levels of agreement with respect to sample size, primary end points, results, and overall interpretations.^10^ However, few preprints are eventually published in journals with the highest impact factors,^10^ and those published by these journals could represent the highest-quality preprints and may be less likely to require major changes during the peer review process. To further inform discourse about the use of preprints in clinical and health science research, we conducted a cross-sectional evaluation examining the agreement between clinical studies posted as preprints to medRxiv in September 2020 and subsequently published in any peer-reviewed journal, including sample size, primary end points, results of primary end points, and overall interpretations.

## Methods

We did not involve members of the public or patients when we designed our study, interpreted the results, or wrote the manuscript. However, we asked members of the public to read our manuscript after submission to ensure it was understandable. The study did not require institutional review board approval because it was based on publicly available information, in accordance with 45 CFR §46. Informed consent was not needed because no patient data were used. This cross-sectional study adheres to the Strengthening the Reporting of Observational Studies in Epidemiology (STROBE) reporting guideline.

### Study Sample

#### Identification of Preprints

To identify a representative sample of clinical and health science preprints, we used medRxiv’s built-in application programming interface (API) to locate all manuscripts posted to medRxiv in September 2020 (eAppendix in Supplement 1). The digital object identifier, preprint title, authors, and medRxiv-assigned study category were automatically collected through the API. Because medRxiv allows authors to post updated versions of their manuscripts, we limited our sample to preprints for which the first posting was in September 2020. For manuscripts that were updated after their initial September 2020 posting, we used medRxiv’s built-in version tracker to locate and select the most recent manuscript version.

#### Preprint Screening

Four investigators (G.J., V.K., X.S., and J.D.W.) manually reviewed each preprint and characterized the study design of each into 1 of the following categories: clinical trials, observational studies, meta-analyses with or without systematic reviews, modeling studies, or other (eAppendix in Supplement 1). One author (J.D.W.) checked 25% of the overall sample for consistency and accuracy. Any disagreements were resolved by consensus.

#### Identification and Timing of Publication

To identify a corresponding peer-reviewed journal article for each preprint (ie, preprint-journal article pairs), we first used the medRxiv API, which matches preprints to journal articles based on title and author(s) (eAppendix in Supplement 1). For preprints that did not have a publication linked with the medRxiv API, we conducted Google searches using the preprint title, key terms, and first and last author(s) names to find corresponding publications. The cutoff date for our publication assessment was September 15, 2022. To measure the time from first posting of a preprint to publication in a peer-reviewed journal, we identified each preprint’s initial date of posting on medRxiv and corresponding e-publication date in a peer-reviewed journal. If a preprint was updated after the initial posting, the date of the most recent version was used for the concordance evaluation, which allowed preprint authors to update their preprint based on internal quality control, community feedback, or any other reason. However, to help ensure that these changes were not conflated with changes made during peer review, we excluded preprints that were updated after the date of journal acceptance. We then used InCites Journal Citation Reports to collect the 2021 journal impact factor for each journal.

#### Evaluation of Agreement Between Preprint-Journal Article Pairs

To evaluate the agreement between preprint-journal article pairs, we narrowed our sample to clinical trials, observational studies, and meta-analyses that measured health-related outcomes. Four investigators (G.J., V.K., X.S., and J.D.W.) reviewed the preprint-journal article pairs and collected the following information: abstract-reported sample size, primary end point(s), results for each primary end point, and overall interpretations or conclusions. For preprints or journal articles with unclear information at the abstract level, we reviewed the full text. For clinical trials and observational studies, sample size was defined as the number of the individuals in the cohort or database used for the primary analysis. For meta-analyses, sample size was defined as the number of discrete studies included in the primary analysis. For each preprint-journal article pair, we identified the measurement scale (eg, odds ratio, mortality rate, or other estimate) and ascertainment time for the primary end points. For clinical trials or prospective observational studies, ascertainment times were defined as the follow-up times for the cohorts of interest. For all other observational study designs and meta-analyses, ascertainment times were defined as the cutoff dates for data collection or database searches. Last, we recorded the Altmetric score for each preprint and journal article.^11^

Using previously developed methods,^10^ we assessed the concordance between the preprint-journal article pairs in terms of sample size, primary end point(s), results of each primary end point, and interpretation. For sample size, the preprint-journal article pairs were classified as concordant if they had numerical equivalence; if a pair was discordant, we conducted further investigation to characterize the type of discordance (eAppendix in Supplement 1).

For primary end point(s), preprint-journal article pairs were classified as concordant if all primary end points identified in the preprint were identified as primary end points in the publication, and no additional primary end points were specified in the publication. For each preprint-journal article pair, results of primary end points were classified as concordant if all effect estimates and/or CIs or *P* values were the same (ie, numerical equivalence). For any results of primary end points classified as discordant, we reviewed the Methods and Results sections of the preprint-journal article pairs to assess the type and potential reasons for the observed discordance (eAppendix in Supplement 1). For the study interpretations, we marked preprint-journal article pairs as concordant if the authors made the same or similar statements about the findings of the study and their implications for health science.

### Statistical Analysis

We conducted descriptive analyses to quantify the preprint characteristics and concordance rates between preprint-journal article pairs. Analyses of concordance rates were repeated across study designs (observational studies, clinical trials, or meta-analyses), journal impact factor (≥10 vs <10), topic (COVID-19–related vs non–COVID-19–related manuscripts), and use of the original posted preprint for the concordance assessment between preprint-journal article pairs with multiple versions. We also abstracted median (IQR) time to publication (in months) from the original post. The χ^2^ test and the Fisher exact test were used to compare concordance rates across COVID-19 relation and journal impact factor. A 2-sided *P* < .05 was considered to be statistically significant. Analyses were conducted using JMP Pro software, version 15.0.0 (SAS Institute Inc), and figures were generated using Python software, version 3.7 (BioVenn package; Python Software Foundation).^12^

## Results

### Preprint Characteristics

**Figure 1.**
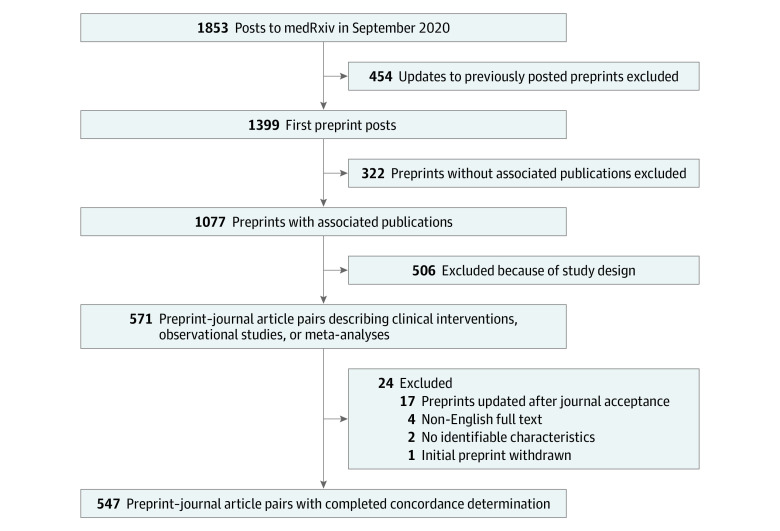
Study Flowchart

We identified 1853 preprints posted on medRxiv in September 2020, of which 1399 (75.5%) were new manuscripts rather than updated versions of previously posted preprints (Figure 1). Of the 1399 preprints, 623 (44.5%) were observational studies, 280 (20.0%) were modeling studies, 62 (4.4%) were meta-analyses with or without systematic reviews, 42 (3.0%) were clinical trials, and 392 (28.0%) were other study designs (Table 1). The most common subject areas were infectious disease (343 [24.5%]), epidemiology (282 [20.2%]), and public and global health (130 [9.3%]); overall, 840 preprints (60.0%) were COVID-19 related.

**Table 1.** Characteristics of All Preprints and Preprint-Journal Article Pairs Initially Posted to medRxiv in September 2020

Characteristic	Original preprints, No. (%) (n = 1399)	Preprints with associated journal article, No. (%) (n = 1077)	Preprint-journal article pairs in final analysis, No. (%) (n = 547)
Study design			
Observational studies	623 (44.5)	489 (45.4)	450 (82.3)
Clinical trials	42 (3.0)	35 (3.2)	32 (5.9)
Meta-analyses	62 (4.4)	47 (4.4)	46 (8.4)
Modeling studies	280 (20.0)	210 (19.5)	NA
Other	392 (28.0)	296 (27.5)	NA
Related to COVID-19			
Yes	840 (60.0)	625 (58.0)	293 (53.6)
No	559 (40.0)	452 (42.0)	254 (46.4)
medRxiv Subject area			
Infectious disease	343 (24.5)	263 (24.4)	113 (20.7)
Epidemiology	282 (20.2)	191 (17.7)	76 (13.9)
Public and global health	130 (9.3)	104 (9.7)	62 (11.3)
Genetic and genomic medicine	67 (4.8)	58 (5.4)	29 (5.3)
Neurology	61 (4.4)	52 (4.8)	30 (5.5)
Psychiatry and clinical psychology	52 (3.7)	38 (3.5)	29 (5.3)
No. of preprint versions, mean (range)	1 (1-5)	1 (1-5)	1 (1-5)
Altmetric score, median (IQR)			
Preprints	2.6 (0.8-2.6)	2.6 (1.0-12.0)	3.0 (1.0-13.0)
Journal articles	NA	9.3 (2.3-32.1)	7.0 (1.0-26.0)

Abbreviation: NA, not applicable.

### Publication Characteristics

Of the 1399 preprints, 1077 (77.0%) had been subsequently published in a peer-reviewed journal as of September 15, 2022 (Table 1), including 489 of 623 observational studies (78.5%), 210 of 280 modeling studies (75.0%), 47 of 62 meta-analyses (75.8%), and 35 of 42 clinical trials (83.3%). The most common subject areas were infectious disease (263 [24.4%]), epidemiology (191 [17.7%]), and public and global health (104 [9.7%]); overall, 625 (58.0%) were COVID-19 related.

The overall median time from first preprint posting to journal publication was 6.0 months (IQR, 3.0-8.0 months), which was consistent across study designs (observational studies: median time to publication, 5.0 months [IQR, 3.0-8.0 months]; modeling studies: median time to publication, 6.0 months [IQR, 3.0-8.0 months]; clinical trials: median time to publication, 6.0 months [IQR, 2.8-9.3 months]; meta-analyses: median time to publication, 6.0 months [IQR, 2.5-9.0 months]; and other designs: median time to publication, 6.0 months [IQR, 3.0-8.0 months]).

### Concordance Between Preprint-Journal Article Pairs

A total of 547 preprint-journal article pairs that measured health-related outcomes were included in our concordance analyses (450 observational studies [82.3%], 32 clinical trials [5.9%], and 46 meta-analyses [8.4%]) (Table 1). The most common subject areas were infectious disease (113 [20.7%]), epidemiology (76 [13.9%]), and public and global health (62 [11.3%]). A total of 293 pairs (53.6%) were related to COVID-19. The median Altmetric scores were 3 (IQR, 1-13) for preprints and 7 (IQR, 1-26) for journal articles. Journal impact factors were obtained for 504 pairs (92.1%), and the median was 5.0 (IQR, 3.7-8.4); 400 of 504 articles (79.4%) were published in journals with an impact factor less than 10.

Of the 535 pairs (97.8%) reporting sample sizes in both sources, 462 (86.4%) were concordant (Table 2); sample sizes were larger in the journal articles for 43 (58.9%) of the 73 pairs with discordant sample sizes. Of the 547 pairs reporting primary end points in both sources, 534 pairs (97.6%) had concordant primary end points. Of the 13 pairs (2.4%) with discordant primary end points, 6 had end points that completely changed between the preprint and journal article, 4 had an addition of at least 1 end point to the journal article, and 3 had removal of at least 1 end point from the journal article.

**Table 2.** Concordance Characteristics for Preprint-Journal Article Pairs

Characteristic	Preprint-journal article pairs, No. (%)
Sample size (n = 535)	
Concordant	462 (86.4)
Discordant	73 (13.6)
Larger in preprint	30 (41.1)
Larger in publication	43 (58.9)
Could not be compared	12
Primary end point(s) (n = 547)	
Concordant	534 (97.6)
Discordant	13 (2.4)
Numerical primary end point results (n = 535)	
Concordant	434 (81.1)
Discordant	101 (18.9)
Effect estimates discordant; direction of effect and statistical significance concordant	66 (65.3)
Effect estimates discordant; direction of effect or statistical significance discordant	5 (5.0)
No. of outcome components or No. of associations discordant	17 (16.8)
Discordant primary end points	13 (12.9)
Could not be compared	12
Study interpretation (n = 547)	
Concordant	526 (96.2)
Discordant	21 (3.8)
No. of preprint versions, median (range) (n = 547)	1 (1-5)
No. of preprint comments, median (range) (n = 547)	0 (0-6)
Altmetric score, median (IQR) (n = 547)	
Preprints	3 (1-11.5)
Journal articles	7 (1-20)

Of the 535 pairs with numerical results for the primary end points, 434 (81.1%) were concordant with respect to numerical effect estimates, direction of effect, and statistical significance. Of the 101 pairs with discordant results, 66 (65.3%) had effect estimates that were in same direction and were statistically consistent, 5 (5.0%) had results in which the direction of the effect estimates or statistical significance were discordant, and 17 (16.8%) had results in which the number of outcomes or number of reported outcomes were discordant.

**Figure 2.**
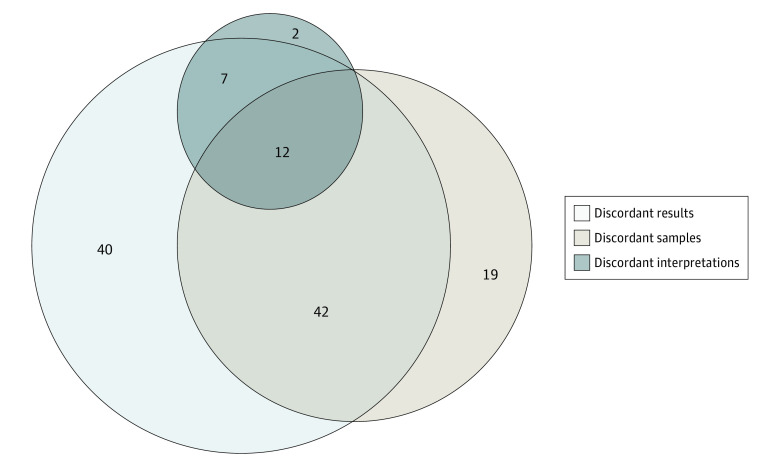
Overlap Between Preprint-Journal Article Pairs With Discordant Sample Sizes, Results, and Interpretations Of the 54 pairs with discordant sample sizes and primary end point results, 13 also had discordant primary end points.

Of the 101 of 535 pairs (18.9%) with discordant results for the primary end points, the most common observed reason for discordance was discordant sample size (46 [45.5%]) (Figure 2; eTable 1 in Supplement 1). A total of 18 pairs (17.8%) had discordant results that were owing to a different number of outcomes or a different number of reported outcome components (ie, association studies), 12 pairs (11.9%) had numerically discordant results for primary end points that were likely owing to minor statistical or methodological changes, and 12 additional pairs (11.9%) had unknown reasons for the discordance of results.

Of the 547 pairs with available study interpretations, 526 (96.2%) had concordant study interpretations, including 82 of 101 pairs (81.2%) with discordant primary end point results (Table 2 and Figure 2). Of the 21 pairs with discordant interpretations, 10 (47.6%) had discordant primary end points (eTable 2 in Supplement 1). Seven additional pairs (33.3%) of the the 21 with discordant interpretations had discordant results for the primary end points. Four pairs (19.0%) had results for primary end points that were concordant, but additional conclusions and interpretations were included in the final journal articles. Overall, 406 of 547 pairs (74.2%) were concordant across all study characteristics: sample size, primary end point, results, and interpretation.

### Concordance Subgroup Analyses

Among pairs with articles published in journals with high impact factors (≥10), the concordance rates were lower than for pairs with articles published in journals with low impact factors for sample size (78 of 103 [75.7%] vs 345 of 391 [88.2%]; *P* = .002) and for primary end point results (66 of 100 [66.0%] vs 331 of 393 [84.2%]; *P* < .001) (Table 3). The concordance rates for primary end points (102 of 104 [98.1%] vs 390 of 400 [97.5%]; *P* ≥ .99) and interpretations (97 of 104 [93.3%] vs 386 of 400 [96.5%]; *P* = .17) were not significantly different between journals with high impact factors and those with low impact factors.

**Table 3.** Concordance Characteristics of Preprint-Journal Article Pairs Published in Journals With High Impact Factors vs Others

Characteristic	Pairs published in journals with impact factor ≥10, No. (%)	Pairs published in journals with impact factor <10, No. (%)	*P* value
Sample size			
No. of pairs	103	391	.002
Concordant	78 (75.7)	345 (88.2)	
Discordant	25 (24.3)	46 (11.8)	
Larger in preprint	7 (28.0)	22 (47.8)	
Larger in publication	18 (72.0)	24 (52.2)	
Could not be compared	1	9	
Primary end point			
No. of pairs	104	400	≥.99
Concordant	102 (98.1)	390 (97.5)	
Discordant	2 (1.9)	10 (2.5)	
Numerical primary end point results			
No. of pairs	100	393	<.001
Concordant	66 (66.0)	331 (84.2)	
Discordant	34 (34.0)	62 (15.8)	
Effect estimates discordant; direction of effect and statistical significance concordant	21 (61.8)	42 (67.7)	
Effect estimates discordant; direction of effect and/or statistical significance discordant	1 (2.9)	4 (6.5)	
No. of outcomes or No. of reported outcomes discordant	10 (29.4)	6 (9.7)	
Discordant primary end points	2 (5.9)	10 (16.1)	
Could not be compared	4	7	
Study interpretation			
No. of pairs	104	400	.17
Concordant	97 (93.3)	386 (96.5)	
Discordant	7 (6.7)	14 (3.5)	

The concordance rates were not significantly different between the 293 pairs reporting results from COVID-19–related studies and the 254 pairs reporting results from non–COVID-19–related studies (eTable 3 in Supplement 1). Concordance rates stratified across study design are available in eTable 4 in Supplement 1.

Finally, we repeated our analysis of concordance using the original versions of preprints. Of the 547 pairs, 87 (15.9%) had preprints with multiple versions. Overall, the concordance rates were relatively consistent when using the original posted preprint instead of the most recent preprint (excluding those updated after journal acceptance) (eTables 5 and 6 in Supplement 1).

## Discussion

In this cross-sectional study of medRxiv preprints of clinical studies subsequently published in peer-reviewed journals, we found that 74.2% were fully concordant with respect to sample size, primary end points, results, and interpretations. When preprint-journal article pairs had discordant results, the discrepancies were often owing to minor sample size changes that were unlikely to affect the study interpretation. Although approximately 25% of preprints are not subsequently published in journals, these findings suggest that among the majority of preprints that are published in journals, changes to studies’ design, results, and conclusions are uncommon. This concordance provides some additional reassurance about the consistency of findings between preprints and subsequently published journal articles.

We found that 81.1% of preprint-journal article pairs were concordant in terms of numerical results of primary end points and that 96.2% were concordant in their interpretations. These findings are in general agreement with a previous study^10^ that focused on the concordance between medRxiv preprints of clinical studies subsequently published in journals with the highest impact factors (>10), in which 68% of results of primary end points and 98% of interpretations were concordant. The current study extends this previous work by examining pairs in all journals, predominantly those with an impact factor less than 10. Our study also mirrors a previous evaluation of preprints posted to bioRxiv and medRxiv, which found that most abstracts did not change significantly after publication.^9^ Similar to our evaluation, these studies also suggest that preprint-journal article pairs with numerical or statistical changes often have consistent conclusions.^9,10^ High levels of concordance have also been observed across other fields, including preprints posted on arXiv and bioRxiv, which are preprint servers for the life and physical sciences, respectively.^13^

Among preprint-journal article pairs with discordant results, we found that most were owing to minor numerical changes or adjustments, likely resulting from updates that increased the study sample size, which did not change the overall interpretations. According to a previous study^14^ examining different versions of preprints and journal articles reporting on COVID-19 interventions, approximately one-third of the pairs with changes in study results had changes in sample size. Although this finding is smaller than our finding, it may not be surprising, given that the previous study examined changes across any of the results, not just the primary end points. Although we found only a few studies with discordant results owing to different primary outcomes or primary outcome components, evidence suggests that outcome switching may be more prevalent among COVID-19–related preprint-journal article pairs.^8,14^ With only 21 pairs with discordant interpretations in our sample, we are unable to draw strong inferences as to why interpretations of preprints change after publication as journal articles. Previous work suggests that preprints within the medical sciences undergo more extensive reframing^15^; however, further research on the potential reasons that preprints change on publication will be necessary.

In the health sciences, the benefits of preprints—namely, rapid dissemination and increased transparency—are typically weighed against concerns that preprint findings are preliminary and subject to change after peer review and editorial oversight.^16,17^ Among our sample of preprints posted 24 months ago (September 2020), less than one-fourth were not published. Although this finding may suggest that there is a sizable fraction of research articles that would not have been publicly available without the opportunity to post as a preprint, the quality and utility of these studies were not assessed. Among preprints that were subsequently published, differences between the published article and the preprint were minor and often owing to changes in sample size, which could be requested by reviewers or editors during peer review. Another study noted that for COVID-19–related clinical trials initially posted as preprints, publication in a peer-reviewed journal did not significantly improve reporting transparency or quality.^18^ Together, these studies may suggest that editors and peer reviewers primarily act as gatekeepers, preventing certain studies from being published and identifying those that merit publication; given the consistency between preprints and publications, perhaps those that merit publication are of higher quality and do not require major changes. However, given that we do not know the final fate of those preprints that have not yet been published, including whether they were ever submitted for publication and/or were rejected after peer review, future evaluations are needed to elucidate the exact role of peer review in preprint evaluation.

### Limitations

This study has several limitations. First, we were able to evaluate only preprints that were published in a peer-reviewed journal. Therefore, we are unable to draw conclusions about preprints that do not make it through the peer review process, whether it be because they were never submitted or were rejected. Future studies could survey authors of preprints without corresponding publications to learn more about the fate of preprints and the role of peer review.^14^ Second, our study was limited to preprints posted in September 2020 and published within 24 months, and additional preprints may eventually be published in subsequent years. However, studies suggest that many manuscripts are published within 7 months of initial submission.^9,14,19,20,21^ Third, given that we captured only preprints that were initially posted to medRxiv in September 2020, it is possible that the types of preprints that are posted may change over time, especially as the COVID-19 pandemic wanes. However, approximately half our sample was of non–COVID-19–related preprints, and we were able to capture a cross-section of preprints posted at a time when the influx of preprints related to COVID-19 leveled off. Fourth, we relied on subjective text review to assess reasons for discordance because not all manuscripts divulge reasons for changes between preprints and published versions of the manuscript. Although automated or algorithmic approaches may be more objective, they would capture only numerical or word changes and not the meanings or interpretations of manuscripts.

## Conclusions

In this cross-sectional study of clinical studies posted as preprints on medRxiv in September 2020 and subsequently published in peer-reviewed journals, most had concordant study characteristics, results, and final interpretations. For preprint-journal article pairs with discordant results, most changes were minor numerical changes, often owing to sample size differences. More than three-fourths of the preprints were published in journals within 24 months, and the results of this study may suggest that most of these preprints report findings that are consistent with the final journal publication.

## Notes

Supplement 1. **eAppendix.** Supplemental Methods and Results **eTable 1.** Observed Reasons for Discordance of Preprint-Journal Article Pairs With Discordant Results for the Primary Endpoint(s), N = 101 **eTable 2.** Observed Reasons for Discordance for Preprint-Journal Article Pairs With Discordant Interpretations, N = 21 **eTable 3.** Concordance Characteristics of COVID-19 vs Non-COVID-19 Preprint-Journal Article Pairs, N = 547 **eTable 4.** Concordance Characteristics for All Preprint-Journal Pairs Across Study Designs, N = 547 **eTable 5.** Concordance Characteristics for Preprint-Journal Article Pairs With Multiple Versions, N = 87 **eTable 6.** Concordance Characteristics for All Preprint-Journal Article Pairs Using the First Posted Version of the Preprint, N = 547
eReferences.

Supplement 2.
Data Sharing Statement
