# Supplementary material for: Comparison of Clinical Study Results Reported in medRxiv Preprints vs Peer-reviewed Journal Articles
Source: JAMA Netw Open. 2022 Dec 9;5(12):e2245847. doi: 10.1001/jamanetworkopen.2022.45847 (PMC9856222; doi:10.1001/jamanetworkopen.2022.45847)
Supplement: Supplement 1. — eAppendix. Supplemental Methods and Results eTable 1. Observed Reasons for Discordance of Preprint-Journal Article Pairs With Discordant Results for the Primary Endpoint(s), N = 101 eTable 2. Observed Reasons for Discordance for Preprint-Journal Article Pairs With Discordant Interpretations, N = 21 eTable 3. Concordance Characteristics of COVID-19 vs Non-COVID-19 Preprint-Journal Article Pairs, N = 547 eTable 4. Concordance Characteristics for All Preprint-Journal Pairs Across Study Designs, N = 547 eTable 5. Concordance Characteristics for Preprint-Journal Article Pairs With Multiple Versions, N = 87 eTable 6. Concordance Characteristics for All Preprint-Journal Article Pairs Using the First Posted Version of the Preprint, N = 547 eReferences. [file jamanetwopen-e2245847-s001.pdf]

Supplementary Online Content

Janda G, Khetpal V, Shi X, Ross JS, Wallach JD. Comparison of clinical study results reported in medRxiv preprints vs peer-reviewed journal articles. *JAMA Netw Open.* 2022;5(12):e2245847. doi:10.1001/jamanetworkopen.2022.45847

eAppendix. Supplemental Methods and Results
eTable 1. Observed Reasons for Discordance of Preprint-Journal Article Pairs With Discordant Results for the Primary Endpoint(s), N = 101
eTable 2. Observed Reasons for Discordance for Preprint-Journal Article Pairs With Discordant Interpretations, N = 21
eTable 3. Concordance Characteristics of COVID-19 vs Non-COVID-19 Preprint-Journal Article Pairs, N = 547
eTable 4. Concordance Characteristics for All Preprint-Journal Pairs Across Study Designs, N = 547
eTable 5. Concordance Characteristics for Preprint-Journal Article Pairs With Multiple Versions, N = 87
eTable 6. Concordance Characteristics for All Preprint-Journal Article Pairs Using the First Posted Version of the Preprint, N = 547
eReferences.

This supplementary material has been provided by the authors to give readers additional information about their work.

eAppendix. Supplemental Methods and Results

Study design classification

Study designs were broken into the following categories based on the following characteristics: clinical trials were studies characterizing the effectiveness of an intervention in a healthcare setting, and were typically associated with a trial registration number; observational studies were prospective, retrospective, or cross-sectional studies using cohort, registry, survey, claims, or electronic health record data; meta-analyses were studies that attempted to examine data from a number of independent studies, and could or could not be associated with a systematic review; modeling studies were studies that involved computational modeling or simulation studies, and typically focused on the accuracy or predictive power of a model; any studies that could not fall into the previous categories (e.g., case reports, protocols) were classified as other.

Identification of preprints using medRxiv

medRxiv links preprints to their corresponding publications by automatically searching for the title and authors, a process that might take up to 2-3 weeks following a publication's online posting and indexing. Authors are also encouraged to link a publication to their preprint by directly corresponding with medRxiv. Along with medRxiv's publication tracking, a manual search (Google) of each preprint title along with the first and last authors was used to find publications that were not identified by medRxiv.

Characterization of discordant sample sizes

For sample sizes classified as discordant, we noted whether the preprint or publication had a larger sample size. If we were unable to determine sample size in either the preprint or publication, we classified the concordance as "could not be determined".

Characterization of discordant results

If a preprint-journal article pair had discordant results that were in the same direction and both results achieved statistical significance, they were classified as "discordant – effect estimates discordant; direction of effect and statistical significance concordant". If a preprint-journal article pair had discordant results of primary endpoints with effect estimates that were not in the same direction or significance, it was marked as "discordant – effect estimates discordant; direction of effect and statistical significance discordant". If a preprint-journal article pair had different endpoints, outcomes, or reported associations they were marked as "discordant – number of outcomes or number of reported outcomes different". If there were no numerical results or no inferential analyses for the primary endpoints, we marked them as "could not be compared".

© 2022 Janda G et al. *JAMA Network Open.*

eTable 1. Observed Reasons for Discordance of Preprint-Journal Article Pairs With Discordant Results for the Primary Endpoint(s), N = 101

**Observed reason for discordance[a]**	**Number (%) of pairs**	**Examples**
Changed primary endpoint	13 (12.9)	*Preprint*: "Aim of this meta-analysis is to evaluate post-operative procalcitonin as a marker to predict post-operative infectious complications after pancreatic surgeries." [1]<br><br>*Publication*: "Aim of this meta-analysis was to compare diagnostic accuracy C-reactive protein and procalcitonin between postoperative days 3 and 5 to predict infectious complications post pancreatic surgery." [2]
Sample size discordance	46 (45.5)	*Preprint*: "Twenty-two competitive athletes with COVID-19 (COVID-19+ athletes) and 44 controls (22 healthy controls and 22 tactical athletes) were included" [3]<br><br>*Publication*: "Fifty-nine COVID-19–positive athletes, 60 athletic controls, and 27 healthy controls were included in our analysis" [4]
Different associations or reported outcome components	18 (17.8)	*Preprint*: "By performing multiple treatment-specific EWAS, we identified 2,894 5'-cytosine-phosphate-guanine-3′ (CpG) sites mapped to 1,583 gene/regions associated with one or more cancer treatments at epigenome-wide significance level (P < 9×10−8)." [5]<br><br>*Publication*: "By performing multiple treatment-specific EWAS, we identified 935 5′-cytosine-phosphate-guanine-3′ (CpG) sites mapped to 538 genes/regions associated with one or more cancer treatments at the epigenome-wide significance level (p < 9 × 10−8)." [6]
Minor statistical/methodological changes	12 (11.9)	*Preprint*: "The Kruskal-Wallis equality-of-populations rank test was used to compare the difference between the medians of the variables during the same time period, i.e. from January to June, in 2018, 2019 and 2020." [7]<br><br>*Publication*: "The Kruskal-Wallis equality-of-populations rank test with Holm-Šídák adjustment was used to compare the difference between the medians of the variables during the same period, that is from January to June, in 2018, 2019, and 2020." [8]

© 2022 Janda G et al. *JAMA Network Open.*

Unclear reason for numerical changes	12 (11.9)	*Preprint:* "Using a random-effects model, comparing e-cigarette users versus non-e-cigarette users, among never-smokers at baseline the odds ratio (OR) for smoking initiation was 3.25 (95%CI 2.61-4.05, I2 85.7%) and among non-smokers at baseline the OR for current smoking was 2.87 (95%CI 1.97-4.19, I2 90.1%)." [9]<br><br>*Publication:* "Using a random-effects model, comparing e-cigarette users versus non-e-cigarette users, among never-smokers at baseline the OR for smoking initiation was 3.19 (95% CI 2.44 to 4.16, I2 85.7%) and among non-smokers at baseline the OR for current smoking was 3.14 (95% CI 1.93 to 5.11, I2 91.0%)." [10]

© 2022 Janda G et al. *JAMA Network Open.*

eTable 2. Observed Reasons for Discordance for Preprint-Journal Article Pairs With Discordant Interpretations, N = 21

**Observed reason for discordance**	**Number (%) of studies**	**Examples**
Different findings in final publication	7 (33.3)	**Preprint**: "Black, Asian and Hispanic ethnic groups are at increased risk of SARS-CoV-2 infection. Black individuals may be more likely to require ICU admission for COVID-19. There may also be disparities in risk of death from COVID-19 at a population level. Our findings are of critical public health importance and should inform policy on minimising SARS-CoV-2 exposure in ethnic minority groups."[11]<br><br>**Publication**: "Individuals of Black and Asian ethnicity are at increased risk of COVID-19 infection compared to White individuals; Asians may be at higher risk of ITU admission and death. These findings are of critical public health importance in informing interventions to reduce morbidity and mortality amongst ethnic minority groups."[12]
Different primary endpoints	10 (47.6)	**Preprint**: "In our meta-analysis we have shown that post-operative PCT is a good indicator to predict post-operative infectious complications after pancreatic surgeries. Tan et al. [5] and cousin et al. [25] had done similar meta-analysis showing use of PCT as a predictor for infectious complications following colorectal surgeries. However, to our knowledge this is the first meta-analysis showing use fullness of post-operative PCT levels in predicting infectious complications following pancreatic surgeries."[1]<br><br>**Publication**: "In our meta-analysis, we evaluated the role of postoperative C-reactive protein and procalcitonin in predicting postoperative infectious complications. Tan et al.5 and cousin et al.34 had done similar meta-analysis showing the use of PCT as a

		predictor for infectious complications following colorectal surgeries. However, to our knowledge, this is the first diagnostic accuracy meta-analysis that simultaneously analysed the role of C-reactive protein (CRP) and procalcitonin (PCT)."[2]
Additions to final publication interpretations (without new findings)	4 (19.0)	**Preprint**: "There were 69 incidences of acute poisoning in children in a single hospital within approximately three years. Most incidents were unintentional and occurred in children younger than 5 years of age. Most poisonings were due to medicines. Analgesics such as paracetamol was the most common documented medicine associated with poisoning. Activated charcoal was the most common treatment. Educating the community and particularly parents about the risks of drugs and chemicals poisoning in children, may reduce the occurrence of such harmful adverse events"[13]<br><br>**Publication**: "Although not common, unintentional and intentional acute poisoning in children does occur. More can be done to legislate and educate parents on safe storage of medicines, household cleaning and other products associated with acute poisoning in children. Likewise, children can be taught more about the risks of poisoning from an early age. As importantly, the number of unclassified patients in this study demonstrates that clinicians need to include detailed notes in the EMR or the system needs to be improved to encourage completeness, to more accurately inform the research evidence-base for future service design, health policy and strategy."[14]

© 2022 Janda G et al. *JAMA Network Open.*

eTable 3. Concordance Characteristics of COVID-19 vs Non-COVID-19 Preprint-Journal Article Pairs, N = 547

**Values are numbers (percentages) unless stated otherwise.**				
**Characteristic**		**Pairs related to COVID-19, N = 293**	**Pairs unrelated to COVID-19, N = 254**	**P-value**
**Sample size**		**N = 284**	**N = 251**	0.61
Concordant		243 (85.6)	219 (87.3)	
Discordant		41 (14.4)	32 (12.7)	
	Larger in preprint	12 (29.3)	18 (56.3)	
	Larger in publication	29 (70.7)	14 (43.8)	
Could not be compared		9	3	
**Primary endpoint**		**N = 293**	**N = 254**	1
Concordant		286 (97.6)	248 (97.6)	
Discordant		7 (2.4)	6 (2.4)	
**Results for primary endpoint results**		**N = 286**	**N = 249**	0.38
Concordant		236 (82.5)	198 (79.5)	
Discordant		50 (7.5)	51 (20.5)	
	Effect estimates discordant; direction of effect and statistical significance concordant	38 (76.0)	28 (54.9)	
	Effect estimates discordant; direction of effect and/or statistical significance discordant	1 (2.0)	4 (7.8)	
	No. of outcomes or No. of reported outcomes discordant	4 (8.0)	13 (25.5)	
	Discordant primary endpoints	7 (14.0)	6 (11.8)	
Could not be compared		7	5	
**Study interpretation**		**N = 293**	**N = 254**	1
Concordant		282 (96.2)	244 (96.1)	
Discordant		111 (3.8)	10 (3.9)	

© 2022 Janda G et al. *JAMA Network Open.*

eTable 4. Concordance Characteristics for All Preprint-Journal Pairs Across Study Designs, N = 547

**Values are numbers (percentages) unless stated otherwise.**			
**Study design**	**Observational studies, N = 467**	**Clinical trials, N = 33**	**Meta-analyses, N = 47**
**Concordance for sample size**	**N = 455**	**N = 33**	**N = 47**
Concordant	398 (87.5)	30 (90.9)	34 (72.3)
Discordant	57 (12.5)	3 (9.1)	13 (27.7)
Larger in preprint	23 (40.4)	1 (33.3)	6 (46.2)
Larger in publication	34 (59.6)	2 (66.7)	7 (53.8)
Could not be compared	12	0	0
**Concordance for primary endpoint**	**N = 467**	**N = 33**	**N = 47**
Concordant	459 (98.3)	32 (97)	43 (91.5)
Discordant	8 (1.7)	1 (3)	4 (8.5)
**Concordance for primary endpoint results**	**N = 457**	**N = 31**	**N = 47**
Concordant	377 (82.5)	24 (77.4)	33 (70.2)
Discordant	80 (17.5)	7 (22.6)	14 (29.8)
Effect estimates discordant; direction of effect and statistical significance concordant	53 (66.3)	4 (57.1)	9 (64.3)
Effect estimates discordant; direction of effect or statistical significance discordant	5 (6.3)	0 (0)	0 (0)
No. of outcome components or No. of associations discordant	14 (17.5)	2 (28.6)	1 (7.1)
Discordant primary endpoints	8 (10.0)	1 (14.3)	4 (28.6)
Could not be compared	10	2	0
**Concordance for study interpretation**	**N = 467**	**N = 33**	**N = 47**
Concordant	451 (96.6)	32 (97.0)	43 (91.5)
Discordant	16 (3.4)	1 (3.0)	4 (8.5)

© 2022 Janda G et al. *JAMA Network Open.*

eTable 5. Concordance Characteristics for Preprint-Journal Article Pairs With Multiple Versions, N = 87

**Values are numbers (percentages) unless stated otherwise.**		
**Characteristic**	**Original preprint-journal article pairs for preprints with update, N = 87**	**Updated preprint-journal article pairs for preprints with update, N = 87**
**Concordance for sample size**	**N = 85**	**N = 85**
Concordant	61 (71.8)	72 (84.7)
Discordant	24 (28.2)	13 (15.3)
Larger in preprint	6 (25.0)	3 (23.1)
Larger in publication	18 (75.0)	10 (76.9)
Could not be compared	2	2
**Concordance for primary endpoint**	**N = 87**	**N = 87**
Concordant	82 (94.3)	85 (97.7)
Discordant	5 (5.7)	2 (2.3)
**Concordance for primary endpoint results**	**N = 80**	**N = 80**
Concordant	51 (63.8)	66 (81.5)
Discordant	29 (36.3)	15 (18.5)
Effect estimates discordant; direction of effect and statistical significance concordant	15 (51.7)	10 (66.7)
Effect estimates discordant; direction of effect or statistical significance discordant	3 (10.3)	1 (6.7)
No. of outcome components or No. of associations discordant	6 (20.7)	2 (13.3)
Discordant primary endpoints	5 (17.2)	2 (13.3)
Could not be compared	7	7
**Concordance for study interpretation**	**N = 87**	**N = 87**
Concordant	74 (85.1)	82 (94.3)
Discordant	13 (14.9)	5 (5.7)

eTable 6. Concordance Characteristics for All Preprint-Journal Article Pairs Using the First Posted Version of the Preprint, N = 547

Characteristic	Number (%)
**Values are numbers (percentages) unless stated otherwise.**	
**Characteristic**	**Number (%)**
**Concordance for sample size, N = 535**	
Concordant	451 (84.3)
Discordant	84 (15.7)
Larger in preprint	33 (39.3)
Larger in publication	51 (60.7)
Could not be compared	12
**Concordance for primary endpoint, N = 547**	
Concordant	531 (97.1)
Discordant	16 (2.9)
**Concordance for primary endpoint results, N = 534**	
Concordant	419 (78.5)
Discordant	115 (21.5)
Effect estimates discordant; direction of effect and statistical significance concordant	71 (61.7)
Effect estimates discordant; direction of effect or statistical significance discordant	7 (6.1)
No. of outcome components or No. of associations discordant	21 (18.3)
Discordant primary endpoints	16 (13.9)
Could not be compared	13
**Concordance for study interpretation, N = 547**	
Concordant	518 (94.7)
Discordant	29 (5.3)
**Dissemination characteristics, N = 547**	
Number of preprint versions, median (range)	1 (1-6)
Number of preprint comments, median (range)	0 (0-6)
**Altmetric score, median (IQR), N = 547**	
Preprints	3 (1-13)
Journal articles	7 (1-26)

© 2022 Janda G et al. *JAMA Network Open.*

eReferences.

1. Vasavada B, Patel H. Post-operative day 3 Procalcitonin predicts post-operative infectious complications in pancreatic surgery – A systemic review and updated meta-analysis. *medRxiv*. 2020:2020.09.21.20198994. doi:10.1101/2020.09.21.20198994
2. Vasavada B, Patel H. Postoperative serum procalcitonin versus C-reactive protein as a marker of postoperative infectious complications in pancreatic surgery: a meta-analysis. https://doi.org/10.1111/ans.16639. *ANZ Journal of Surgery*. 2021/05/01 2021;91(5):E260-E270. doi:https://doi.org/10.1111/ans.16639
3. Clark DE, Parikh A, Dendy JM, et al. COVID-19 Myocardial Pathology Evaluated Through scrEening Cardiac Magnetic Resonance (COMPETE CMR). *medRxiv*. 2020:2020.08.31.20185140. doi:10.1101/2020.08.31.20185140
4. Clark DE, Parikh A, Dendy JM, et al. COVID-19 Myocardial Pathology Evaluation in Athletes With Cardiac Magnetic Resonance (COMPETE CMR). *Circulation*. 2021/02/09 2021;143(6):609-612. doi:10.1161/CIRCULATIONAHA.120.052573
5. Song N, Hsu C-W, Pan H, et al. Persistent Variations of Blood DNA Methylation Associated with Treatment Exposures and Risk for Cardiometabolic Outcomes among Long-term Survivors of Childhood Cancer: A Report from the St. Jude Lifetime Cohort. *medRxiv*. 2020:2020.09.10.20192393. doi:10.1101/2020.09.10.20192393
6. Song N, Hsu C-W, Pan H, et al. Persistent variations of blood DNA methylation associated with treatment exposures and risk for cardiometabolic outcomes in long-term survivors of childhood cancer in the St. Jude Lifetime Cohort. *Genome Medicine*. 2021/04/06 2021;13(1):53. doi:10.1186/s13073-021-00875-1
7. Dong Q, Kuria A, Weng Y, Liu Y, Cao Y. Impacts of the COVID-19 epidemic on the department of stomatology in a tertiary hospital: a case study in the General Hospital of the Central Theater Command, Wuhan, China. *medRxiv*. 2020:2020.09.11.20192450. doi:10.1101/2020.09.11.20192450
8. Dong Q, Kuria A, Weng Y, Liu Y, Cao Y. Impacts of the COVID-19 epidemic on the department of stomatology in a tertiary hospital: A case study in the General Hospital of the Central Theater Command, Wuhan, China. https://doi.org/10.1111/cdoe.12680. *Community Dentistry and Oral Epidemiology*. 2021/12/01 2021;49(6):557-564. doi:https://doi.org/10.1111/cdoe.12680
9. Baenziger ON, Ford L, Yazidjoglou A, Joshy G, Banks E. E-cigarette use and combustible tobacco cigarette smoking uptake among non-smokers, including relapse in former smokers: umbrella review, systematic review and meta-analysis. *medRxiv*. 2020:2020.09.16.20195438. doi:10.1101/2020.09.16.20195438
10. Baenziger ON, Ford L, Yazidjoglou A, Joshy G, Banks E. E-cigarette use and combustible tobacco cigarette smoking uptake among non-smokers, including relapse in former smokers: umbrella review, systematic review and meta-analysis. *BMJ Open*. 2021;11(3):e045603. doi:10.1136/bmjopen-2020-045603
11. Sze S, Pan D, Gray LJ, et al. Ethnicity and clinical outcomes in COVID-19: A Systematic Review and Meta-analysis. *medRxiv*. 2020:2020.09.05.20188821. doi:10.1101/2020.09.05.20188821
12. Sze S, Pan D, Nevill CR, et al. Ethnicity and clinical outcomes in COVID-19: A systematic review and meta-analysis. *eClinicalMedicine*. 2020;29doi:10.1016/j.eclinm.2020.100630
13. Tobaiqy M, Asiri BA, Sholan AH, et al. Frequency and Management of Drug and Chemical Poisoning among Children Attending an Emergency Department in a Single Hospital in Saudi Arabia. *medRxiv*. 2020:2020.09.08.20190868. doi:10.1101/2020.09.08.20190868

© 2022 Janda G et al. *JAMA Network Open.*

14. Tobaiqy M, Asiri BA, Sholan AH, et al. Frequency and Management of Acute Poisoning Among Children Attending an Emergency Department in Saudi Arabia. *Pharmacy*. 2020;8(4):189.

© 2022 Janda G et al. *JAMA Network Open.*
