# Supplementary material for: Comparison of Clinical Study Results Reported in medRxiv Preprints vs Peer-reviewed Journal Articles
Source: JAMA Netw Open. 2022 Dec 9;5(12):e2245847. doi: 10.1001/jamanetworkopen.2022.45847 (PMC9856222; doi:10.1001/jamanetworkopen.2022.45847)
Supplement: Supplement 2. — Data Sharing Statement [file jamanetwopen-e2245847-s002.pdf]

Data Sharing Statement

Janda. Comparison of Clinical Study Results Reported in medRxiv Preprints vs Peer-Reviewed Journal Articles. *JAMA Netw Open*. Published December 09, 2022.
doi:10.1001/jamanetworkopen.2022.45847

Data
Data available: Yes
Data types: Data (not involving human participants)
How to access data: joshua.wallach@emory.edu
When available: With publication

Supporting Documents
Document types: None

Additional Information
Who can access the data: Anyone requesting the data
Types of analyses: for any purpose
Mechanisms of data availability: An excel file will be provided
